# Analysis on the Vulnerability of a Tunnel Entrance under Internal Explosion

**DOI:** 10.3390/s22249727

**Published:** 2022-12-12

**Authors:** Zichao Liu, Jun Wu, Qinyi Chen, Shutao Li, Qiushi Yan, Haitao Yu

**Affiliations:** 1 School of Urban Rail Transportation, Shanghai University of Engineering Science, Shanghai 201620, China; 2 Institute of Defense Engineering, AMS, PLA, Beijing 100036, China; 3 Key Laboratory of Urban Security and Disaster Engineering, Ministry of Education, Beijing University of Technology, Beijing 100124, China; 4 Department of Geotechnical Engineering, Tongji University, Shanghai 200092, China

**Keywords:** tunnel entrance, internal explosion, residual bearing capacity, vulnerability analysis

## Abstract

Tunnels play an essential role in the transportation network. Tunnel entrances are usually buried at a shallow depth. In the event of an internal explosion, the blast pressure will cause severe damage or even collapse of the tunnel entrance, paralyzing the traffic system. Therefore, an accurate assessment of the damage level of tunnel entrances under internal blast loading can provide effective assistance for the anti-blast design of tunnels, post-disaster emergency response, and economic damage assessment. In this paper, four tunnel entrance specimens were designed and fabricated with a scale ratio of 1/5.5, and a series of field blast tests were carried out to examine the damage pattern of the tunnel entrances under internal explosion. Subsequently, static loading tests were conducted to obtain the maximum bearing capacity of the intact specimen and residual bearing capacities of the post-blast specimens. After that, an explicit non-linear analysis was carried out and a numerical finite element (FE) model of the tunnel entrance under internal blast loading was established by adopting the arbitrary Lagrangian–Eulerian (ALE) method and validated based on the data obtained from the field blast and static loading tests. A probabilistic vulnerability analysis of a typical tunnel entrance subjected to stochastic internal explosions (assuming various charge weights and detonation points) was then carried out with the validated FE model. For the purpose of damage assessment, the residual bearing capacity of the tunnel entrance was taken as the damage criterion. The vulnerability curves corresponding to various damage levels were further developed based on the stochastic data from the probabilistic vulnerability analysis. When the charge weight was 200 kg, the tunnel entrance exhibited slight or moderate damage, while the tunnel entrance suffered severe or even complete damage as the charge weight increased to 1000 kg. However, the tunnel entrance’s probability of complete damage was less than 10% when the TNT charge weight did not exceed 1000 kg.

## 1. Introduction

In recent decades, terrorist attacks have occurred frequently around the world [1,2,3]. As an important infrastructure in the transportation system, tunnels have become a key target for terrorist attacks. In addition, accidental explosions caused by negligence during transportation frequently occur. The occurrence of terrorist attacks and accidental explosions may bring a serious threat to the tunnel infrastructure, seriously endangering the safety of people’s lives and property. Hence, it is of great significance to investigate the dynamic response of tunnels under internal blast loading and assess the damage level of tunnels after explosions.

Due to the semi-enclosed space of the tunnel, the blast wave generated by internal explosion will experience multiple reflections and superimpositions, and therefore the transmission of the blast wave within tunnels is very complicated compared to that of a free field explosion [4]. In recent years, many scholars have conducted experiments and numerical simulations of tunnels under blast loads [5,6,7,8,9,10,11]. Meng et al. [12] conducted a field test on a tunnel with dimensions of 20,000 × 1800 × 600 mm under a gas explosion. The results demonstrated that the tunnel linings were prone to cracking due to the uneven pressure distribution in the semi-enclosed space. Tiwari et al. [11] conducted numerical simulations of tunnels under internal blast loads. The influence of three parameters, namely lining thickness, explosive equivalent and friction angle, on the dynamic response of the tunnel was discussed. The results showed that the deformation of the tunnel lining became more severe with increasing charge weight and decreased with increasing lining thickness or shear strength of the surrounding soil layer [13]. Xu et al. [14] conducted internal explosion tests and numerical simulations on a scaled immersed tunnel model. The results showed that the major damage was concentrated at the mid-span and corners of the tunnel roof. Yang et al. [15] carried out numerical simulations on underwater tunnels under internal explosion conditions and proposed four damage levels based on the deflection-span ratio criterion, i.e., slight damage, moderate damage, severe damage and collapse. Afterwards, a damage assessment model for underwater tunnels after an internal explosion was established. It should be noted that tunnel entrances are normally constructed at shallow burial depths. For shallow-buried tunnels under internal blast loads, the blast wave propagates in a spherical pattern, which might cause bending or collapse failure of the tunnel [16,17]. However, there are limited studies on the blast performance of tunnel arches under the conditions of internal explosions. Liu et al. [13] found that for an internal explosion, the lower the thickness of the ground above the tunnel, the more severe the damage to the tunnel. Choi et al. [16] numerically investigated the anti-blast performance of tunnels under the conditions of internal explosion with TNT charges varying from 230 to 910 kg. The results showed that compared to tunnels with deep burial depths, shallow-buried tunnels were more vulnerable to internal explosions. Therefore, it can be seen that under an internal explosion, shallow-buried tunnels (i.e., the tunnel entrance) might be destroyed by intense blast loading, thereby causing the collapse of the tunnel entrance or the surrounding ground. Therefore, it is crucial to analyze the dynamic response of the tunnel entrance and to assess the damage level of the tunnel entrance under different magnitudes of internal blast loading.

Vulnerability analysis is a very mature assessment method in the field of earthquake engineering [18,19]. In the vulnerability analysis of tunnels subjected to seismic loading, vulnerability curves can be established to comprehensively assess the seismic performance of tunnels by selecting vibration strength indicators and engineering parameters [20]. Methods of analysis such as seismic damage investigation, numerical simulation and experimental simulation are usually adopted [21,22,23,24,25]. Hazus-MR4 [21] collected data on the damage to tunnels in earthquakes around the world and further used such data to classify the degree of seismic damage of tunnels into intact, minor, moderate, severe and complete damage. The vulnerability curves of different types of tunnels under various peak vibrations were also developed. Avanaki et al. [24] investigated the effect of steel fiber reinforced lining on the seismic vulnerability of a tunnel by employing nonlinear quasi-static seismic analysis. Argyroudis et al. [20] proposed a numerical method to construct the vulnerability curves of shallow buried tunnels under seismic loading by considering the uncertainties of the shape of the tunnel section. However, there have been very few vulnerability analyses of tunnels under blast loads. Choi et al. [16] studied the dynamic response of tunnels under internal explosion by considering the factors of explosive mass, distance, and tunnel shape. Then, damage assessment diagrams of tunnels were developed, which provided important data for vulnerability assessment of tunnels under blast loads. Chaudhary et al. [26] established an FE model of a tunnel with a diameter of 5 m. A probabilistic dynamic analysis of the tunnel with different lining materials was then carried out, and the probability of failure curve was obtained for the tunnel at different explosive equivalents.

In summary, the application of the vulnerability method to the seismic analysis of tunnels can not only adequately take the stochasticity and uncertainty of earthquakes into account but can also truly reflect the damage of tunnels. In addition, the damage levels of tunnels subjected to earthquakes can be predicted. Although the vulnerability analysis of tunnels is widely applicable in seismic engineering, it is still rarely seen in the field of explosions. Therefore, how to reasonably apply vulnerability analysis to tunnels under blast loads and determine an appropriate damage criterion to study the damage of tunnels needs to be further studied. Therefore, in this paper, a high-fidelity FE model of the tunnel entrance was established by using the arbitrary Lagrangian–Eulerian (ALE) method. The results of the field blast test and static loading test were employed to verify the accuracy of the developed FE model. Then, a prototype model of the tunnel entrance was established based on the validated FE model. A probabilistic vulnerability analysis of the tunnel entrance subjected to internal explosions was further conducted with consideration of the uncertainty of explosive resources (i.e., charge weights and detonation points). For the purpose of the damage assessment, the residual bearing capacity of the tunnel entrance was taken as the damage criterion. The vulnerability curves corresponding to various damage levels were developed based on stochastic data from probabilistic vulnerability analysis. This study contributes to the anti-blast design of tunnel structures and further provides a new research perspective on the vulnerability of tunnel entrances under internal explosions.

## 2. Validation of the FE Model

### 2.1. Field Blast Test and Static Test

In this paper, a total of four tunnel entrance models were fabricated, three of which were tested in the field blast test, and one of which was adopted for the static loading test. The design of the model was based on a prototype of a tunnel entrance with a scale ratio of 1:5.5. The blast load was described by using the cube root scaling rule [27]. For the tunnel entrance specimen, it was first assumed that the same materials were used for the model and the prototype. Furthermore, to reduce the effect of dimensions on the dynamic response of the tunnel structure, the same reinforcement ratio was adopted in the model, and the inner and outer diameter ratios were kept the same as those in the prototype. Dimensional analysis was then used to derive material properties (i.e., compressive strength and concrete strain) as well as structural properties (i.e., length volume and structural mass). The similarity factor of the tunnel entrance model in the present study is reported in Table 1 [28,29].

**Figure 1:**
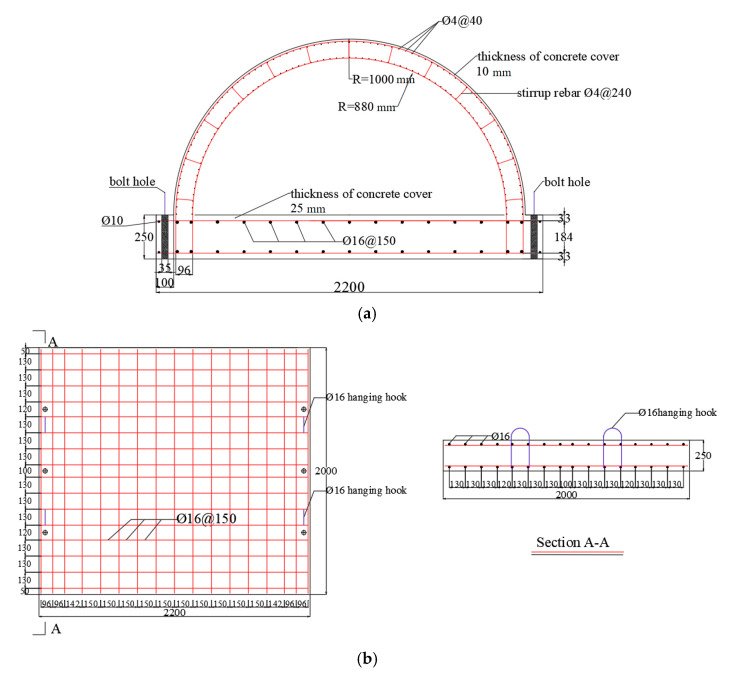
Geometrical dimensions and reinforcement of the specimen (unit: mm): (**a**) Tunnel; (**b**) Foundation slab.

Because the present study mainly focuses on the damage to the tunnel crown and hance of the tunnel entrance under internal explosion, the curved wall and invert of the tunnel were simplified as the foundation slab. Therefore, the tunnel structure was directly connected with the foundation slab in the tunnel entrance model. The longitudinal length of the specimen was 2000 mm, while the outer and inner radii of the tunnel arch were 1000 mm and 880 mm, respectively. The thickness of the tunnel lining was 120 mm. The ratio of the buried depth to the tunnel diameter was kept at 0.15; therefore, the depth of the covering soil layer was 300 mm in the test [30]. The concrete strength of the tunnel lining was 31.35 MPa [31]. Double layers of 6 mm diameter rebar were spaced 85 mm apart both in circumferential and longitudinal directions in the tunnel lining, and the concrete cover of the lining was 10 mm. The yield strength of the rebar was 450 MPa. The rebar of the tunnel lining was connected to the foundation slab, which had a thickness of 250 mm and was cast by premixed concrete with a compressive strength of 45.65 MPa. Rebar with a 16 mm diameter was arranged in the foundation slab. The geometrical dimensions and the reinforcement of the tunnel entrance specimen are illustrated in Figure 1.

A total of three detonation positions were designated to investigate the dynamic response of the tunnel entrance under internal blast loading. The detonation points were the center of the tunnel, the tunnel crown and the hance of the tunnel. The detonation points for all blast scenarios were set at the middle of the specimen along the longitudinal direction. The field blast test program is listed in Table 2.

**Figure 2:**
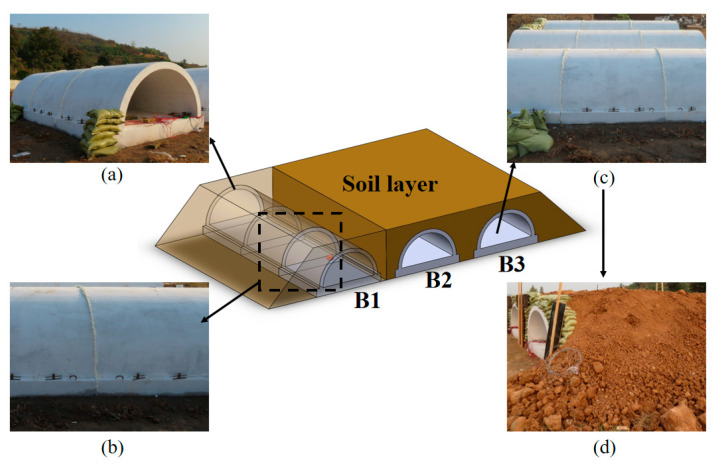
Test setup: (**a**) Scene photo of the tunnel specimens; (**b**) Details of foam filling; (**c**) Prepare for covering soil; (**d**) Finish covering.

In the field blast test, each tunnel entrance model was connected to an additional tunnel section, in which the entrance model was placed at one end, as shown in Figure 2. Therefore, during the internal explosion, the blast wave could propagate along the longitudinal direction of the tunnel section. The gap between the specimen and the additional tunnel section was filled by foam to reduce the influence of reflection of the blast wave on the specimen.

**Figure 3:**
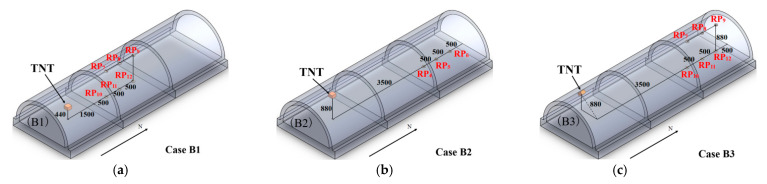
Layout of reflected overpressure sensors (unit: mm): (**a**) Case B1; (**b**) Case B2; (**c**) Case B3.

In the test, overpressure sensors were used to record the reflected overpressure for each shot. The overpressure sensors were located at the additional tunnel section. The layout diagram of overpressure sensors for each case is shown in Figure 3.

**Figure 4:**
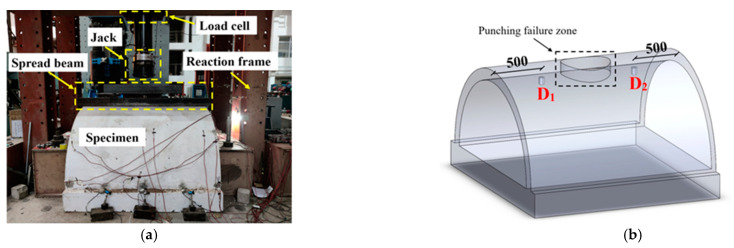
Test setup: (**a**) Scene photo; (**b**) Layout of displacement sensors (unit: mm).

After the field blast test, the three blast-damaged specimens were transferred to the laboratory, along with one intact specimen for the static loading test. The setup of the static load test is given in Figure 4a. A spread beam was arranged on the top of the tunnel crown and contacted with the hydraulic servo jack, thus transferring the vertical load uniformly to the specimen. Two displacement sensors were employed at the inner side of the tunnel crown, which were used to measure the deflection of the specimen during the static tests, as shown in Figure 4b. The test program of the static loading test is listed in Table 3.

### 2.2. Development of the FE Model

#### 2.2.1. Spatial Discretization and Modeling Process

**Figure 5:**
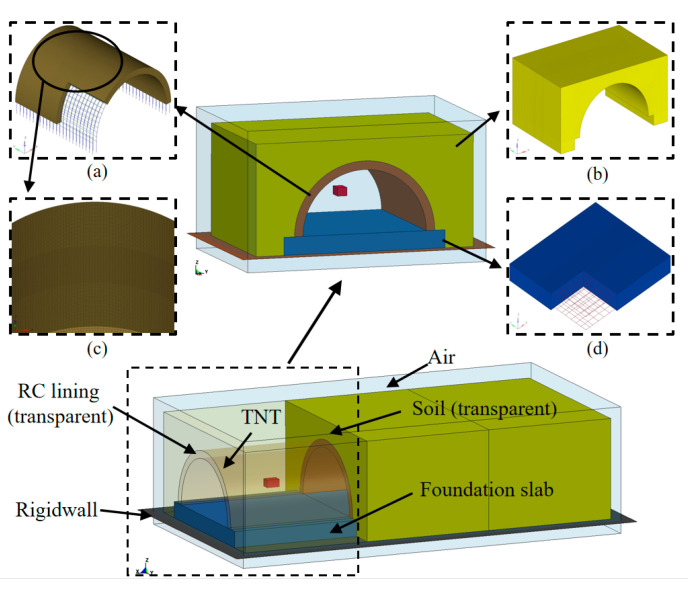
FE model of the blast test: (**a**) RC lining; (**b**) Soil layer; (**c**) Details of RC lining; (**d**) Foundation slab.

In this study, explicit non-linear analyses were conducted, in which a finite element (FE) model was established and solved via the commercial software LS-DYNA. In LS-DYNA, the element of the Lagrangian algorithm is attached to the material, and the deformation of the element is generated with the flow of the material. However, when the deformation of the structure is enormous, it may lead to serious distortion of the finite element mesh, which will result in an invalid calculation. The Eulerian algorithm is based on spatial coordinates, and the element is fixed in space during the analysis which will not move with the component. The arbitrary Lagrangian–Eulerian (ALE) algorithm has the advantages of both the Lagrangian and Eulerian algorithm [32,33,34,35]. The elements can move independently of the material and spatial coordinate systems, which can avoid serious distortion. In addition, it can effectively track the motion of the structure boundary and maintain a reasonable shape of the elements, which is widely adopted for solving large deformation issues in the field of structural explosion [36,37,38]. Hence, the ALE algorithm was employed to simulate the interaction between the blast wave and the tunnel entrance. As shown in Figure 5, the FE model consisted of the soil layer, RC lining, foundation slab, rebar, air and TNT charge. Lagrangian algorithms were used to describe solid materials such as soil layer, RC lining, foundation slab. Eulerian algorithms were utilized for air and TNT charge components. The interaction between Eulerian and Lagrangian elements was attained using the keyword * CONSTRAINED_LAGRANGE_IN_SOLID. A face-to-face contact algorithm was employed to simulate the interaction between the lining and the soil. The effect of the initial earth stress on the buried tunnel entrance was considered by applying gravity to the soil layer via the keyword * LOAD_BODY_Z. The rebar was modeled by Hughes-Liu beam elements and connected into the concrete with the keyword * CONSTRAINED_LAGRANGE_IN_SOLID. After a mesh convergence study, a mesh size of 15 mm was determined for the center of the RC lining, and a mesh size of 20 mm was determined for the rest of the Lagrangian models. In addition, the Eulerian mesh size was determined to be 10 mm in the FE model [39,40]. The erosion algorithm was adopted in the FE simulation. After several preliminary simulations, a maximum tensile strain erosion criterion with a value of 0.1 was used in this study [41,42].

**Figure 6:**
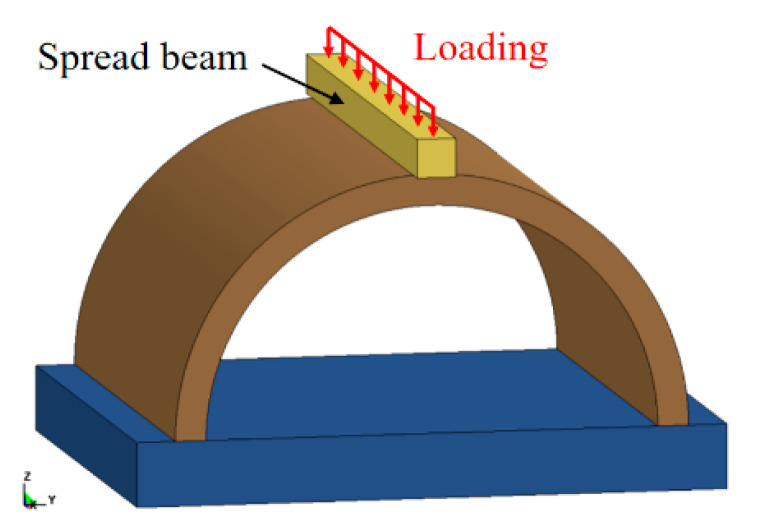
FE model of the undamaged tunnel under static loading.

**Figure 7:**
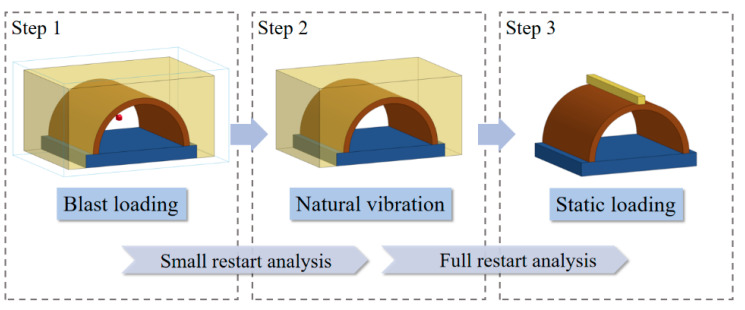
Process of calculation.

Figure 6 shows the FE model of the intact specimen under static loading. For the FE model of static loading, the spread beam vertically moved to the tunnel crown with a constant velocity of 0.1 mm/ms. In addition, to obtain the residual bearing capacity of the blast-damaged specimens, it was necessary to first apply a small restart after the explosion simulation. The aim was to allow the blast-damaged specimen to vibrate freely. When the global system stabilized, a full restart function in LS-DYNA was employed to connect the explosion and static loading phases. It should be noted that dynamic explicit analysis was employed in all phases. The simulation process for the calculation of the residual bearing capacity of the blast-damaged specimen is shown in Figure 7.

#### 2.2.2. Material Model

##### Concrete and Rebar

In this study, the * MAT_CONCRETE_DAMAGE_REL3 model (i.e., the KCC model) was employed to simulate the dynamic response of the tunnel [43,44,45]. Three independent strength surfaces (i.e., the yield strength surface, maximum strength surface, and residual strength surface) are defined in the KCC model to fully represent the elastic, plastic and softening behaviors of the concrete-like materials being stressed. The parameters for the strength surface are *a*_0_, *a*_1_, *a*_2_, *a*_0y_, *a*_1y_, *a*_2y_, *a*_1f_ and *a*_2y,_ which can be determined by triaxial compressive or tensile tests. At the same time, users only need to input the uniaxial compressive strength and Poisson’s ratio of the concrete, and then other parameters can be automatically generated. However, in the KCC model, the default compressive and tensile softening parameters (*b*_1_ and *b*_2_) are only valid for an element size of 25.4 mm and thus should be modified if different element sizes are used in the FE simulation [46]. For the concrete with a cylindrical strength of 24.7 MPa, *G_c_* was 13.62 MPa * mm, the area under the post-peak stress-strain curve *G_c_*/*h* was 0.91 and the corresponding *b*_1_ was 3.11 after calculation. In the same way, *b*_2_ was 1.25. For the concrete with a cylindrical strength of 37.4 MPa, *G_c_* was 14.38 MPa * mm, the area under the post-peak stress-strain curve *G_c_*/*h* was 0.96 and the corresponding *b*_1_ was 2.73 after calculation. In the same way, *b*_2_ was 1.32. Table 4 lists the key parameters of the KCC model utilized in the current study. The equation of state 8 (* EOS_TABULATED_COMPACTION) was employed in the KCC model to describe the relationship of volumetric strain and bulk modulus under various hydrostatic pressures.

The * MAT_PLASTIC_KINEMATIC material model was utilized to characterize the dynamic behavior of the rebar. The model is related to the strain rate of the material and is very suitable for the simulation of isotropic and kinetic plastic hardening materials. In this paper, the yield strength of the rebar is 450 MPa, the elastic modulus is 200 GPa, and the strain rate parameters *C* and *P* are 40 s^−1^ and 5, respectively. In addition, the failure strain of the rebar was set to 0.15, which meant that beam elements would be deleted if their strain value exceeded 0.15, indicating rupture of the rebar.

##### Soil Layer

In the current study, the soil layer was simulated by the * MAT_SOIL_AND_FORM material model [47,48,49], in which the stress yield function can be expressed as:

(1) $$\phi_{S}=S_{ij}s_{ij}/2-\left[a_{0}+a_{1}p+a_{2}p^{2}\right]$$

where *a*_0_, *a*_1_ and *a*_2_ are yield function constants, which can be determined by the internal friction angle *φ* and soil cohesion *C*. *S_ij_* denotes the deviatoric stress tensor. *p* is the hydrostatic pressure, and the relationship with the volume strain is shown in Table 5 [50]. Based on the measured data of the soil in the field blast test, the main parameters of the soil in the FE model are shown in Table 6.

##### Air and TNT

The air in the FE model was considered an inviscid ideal gas, which was represented by the * MAT_NULL material model. The keyword * EOS_LINEAR_POLYNOMIAL was used to simulate the equation of state of the air, which is expressed as [50]:

(2) $$P=C_{0}+C_{1\mu }+C_{2\mu }^{2}+C_{3\mu }^{3}+\left(C_{4}+C_{5\mu }+C_{6\mu }^{2}\right)E$$

(3) μ=1/V−1

where *P* is the pressure, *E* represents the internal energy per unit volume, *V* is the initial relative volume, and *C*_0_, *C*_1_, *C*_2_, *C*_3_, *C*_4_, *C*_5_, and C_6_ are the coefficients of the polynomial equation; for ideal air, *C*_0_ = *C*_1_ = *C*_2_ = *C*_3_ = *C*_6_ = 0 and *C*_4_ = *C*_5_ = 0.4 [33,38,50].

The TNT charge was described as * MAT_HIGH_EXPLOSIVE_BURN, and its corresponding equation of state was simulated by * EOS_JWL. The *P*-*V* relation of the JWL equation of state is expressed as follows [50]:

(4) $$P=A\left(1-\frac{\omega }{R_{1}v}\right)e^{-R_{1}V}+B\left(1-\frac{\omega }{R_{2}V}\right)e^{-R_{2}V}+\frac{\omega E_{0}}{V}$$

where *P* is the detonation pressure, *V* is the initial relative volume, *E*_0_ is the detonation energy per unit volume, and *ω, A, B, R*_1_ and *R*_2_ are polynomial equation coefficients. The key parameters of air and TNT charge in the current study are shown in Table 7.

### 2.3. Validation

#### 2.3.1. Reflected Overpressure

**Figure 8:**
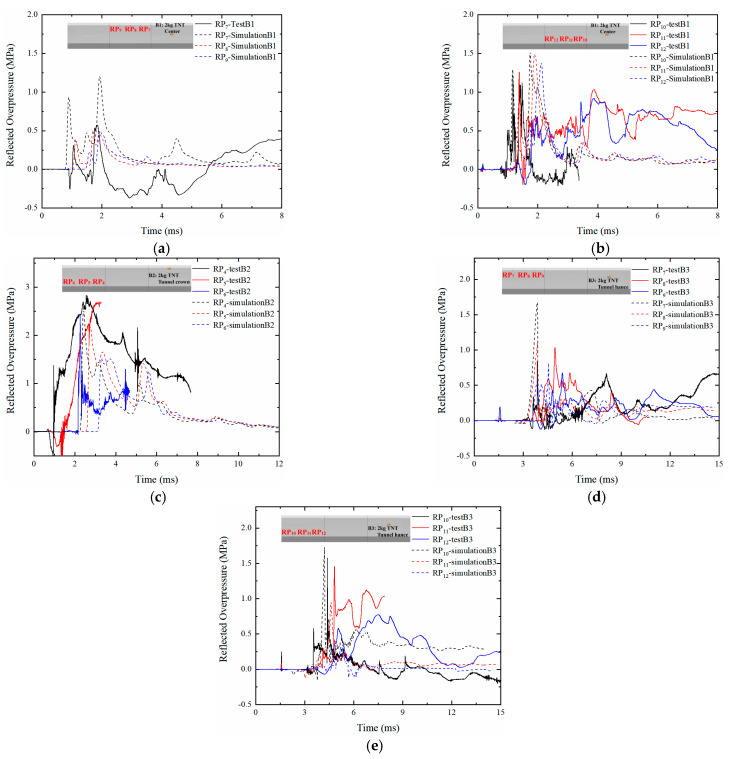
Time histories of reflected overpressure: (**a**,**b**) Case B1; (**c**) Case B2; (**d**) and (**e**) Case B3.

Figure 8 reports the history curves of the reflected overpressure from the field blast test and FE simulation. The arrival time of the blast wave from the FE simulation was generally close to that of the field blast test. In addition, the FE simulation predicted that the attenuation rate of the overpressure was intense with several peaks, which is consistent with the experimental results. Table 8 reports the comparison of the peak-reflected overpressure from the field blast test and the FE simulation. It is shown that most of the peak-reflected overpressure predicted by the FE simulation is in good agreement with the experimental results, in which the deviations were less than 30%. However, for RP_7_ in Case B3, there was a large deviation for the peak-reflected overpressure between the experimental and FE simulations. This may be due to the decrease in soil mass affecting the sensors during the field blast test.

#### 2.3.2. Damage Pattern

**Figure 9:**
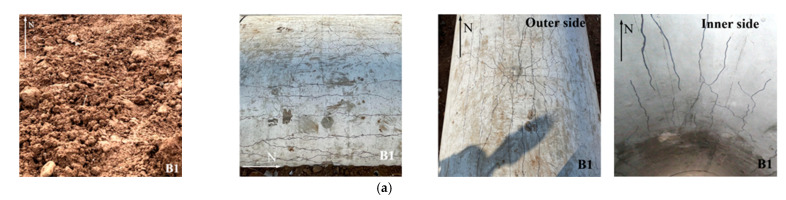

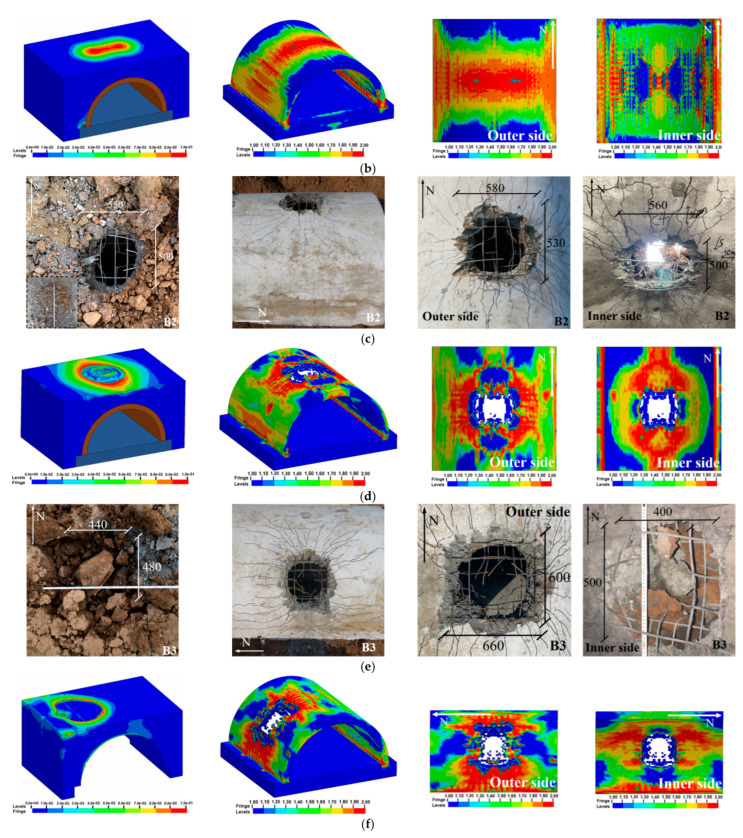
Experimental and simulated damage pattern of the specimens under explosion load (unit: mm): (**a**) Case B1-test; (**b**) Case B1-simulatoin; (**c**) Case B2-test; (**d**) Case B2-simulatoin; (**e**) Case B3-test; (**f**) Case B3-simulatoin.

In the presentation of the FE results, the effective plastic strain (EPS) was used to reflect the damage to the tunnel entrance. Since the current research focused on the damage pattern of the specimen after an internal explosion, the post peak behavior of the tunnel entrance was of great interest. Thus, the EPS was selected to vary from 1 to 2, which corresponded to the concrete material deteriorating from its maximum strength to its residual strength [46,52]. Figure 9 presents the damage pattern of the tunnel entrance under the internal explosion from the FE simulation. Under the same TNT charge weight, the punching failure caused by the contact detonation at the tunnel hance was greater than that at the tunnel crown, which was consistent with the field blast test results. The comparison of the punching failure zone between the experimental and FE results is further reported in Table 9. The dimensions of the punching failure zone in the two specimens were close to the test result, and the maximum deviation between the FE simulation and experimental result was less than 10%, indicating the accuracy of the developed FE model. In the FE simulation, all the specimens suffered tensile damage at the tunnel foot, which was consistent with the field observation. Figure 9 also reports the deformation of the soil layer above the tunnel. Note that the plastic strain was used to present the deformation of the soil layer in the FE simulation. The soil layer above the tunnel entrance exhibited perforation due to contact detonation, while a small settlement of the soil layer was observed for the tunnel after the center explosion. These phenomena were also observed in the field blast test.

#### 2.3.3. Residual Bearing Capacity

The maximum bearing capacity of the specimen predicted by the FE simulation is given in Table 10. The maximum bearing capacity of the intact specimen from the FE simulation was 585 kN, which had a deviation of 2.5% from the experimental result. Table 10 further reports the comparison of the test and FE simulation for the residual bearing capacity of the blast-damaged specimens. The deviations between the static loading test and FE simulation for each specimen were 2.5%, 3.9%, 9.3% and 11.5%, demonstrating the precision of the FE model developed in this study. In addition, for the same TNT charge weight, the specimen under the condition of contact detonation at the tunnel hance exhibited a greater residual bearing capacity than that under the condition of contact detonation at the tunnel crown, which was consistent with the experimental results. This phenomenon further demonstrates the accuracy of the FE model and modeling process in the current study.

## 3. Prototype of the Tunnel Entrance under Internal Explosion

### 3.1. FE Model

**Figure 10:**
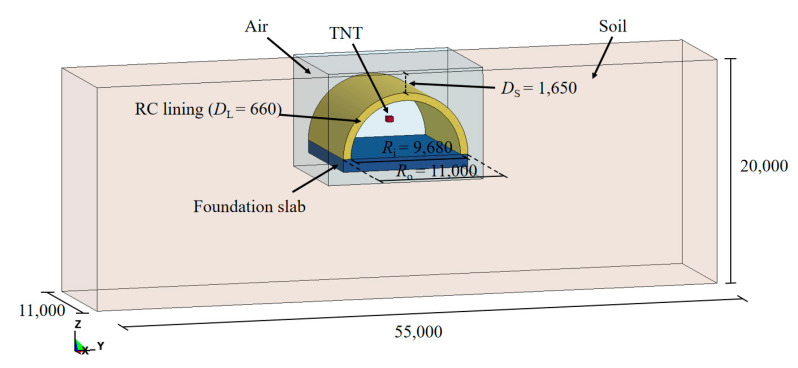
FE model of the prototype of tunnel entrance (unit: mm).

Based on the above validated material model and modeling algorithm, the FE model of the tunnel entrance was scaled up to the prototype of the tunnel entrance with a scale ratio of 1:5.5, as shown in Figure 10. In the FE model of the prototype of the tunnel entrance, the outer diameter *R*_o_ and inner diameter *R*_i_ of the RC lining were 11 m and 9.68 m, respectively, and, thus, the lining thickness *D*_L_ was 0.66 m. A diameter (*d*) of 24 mm was utilized at both double layers of the bidirectional rebars. The dimensions of the surrounding soil layer were 55,000 × 11,000 × 20,000 mm (length × width × height), and the buried depth (*D*_s_) was determined to be 1.65 m. The mesh sizes for the key components in the FE model of the prototype of the tunnel entrance were consistent with those verified in Section 2.2.1 (i.e., a mesh size of 15 mm was used for the center of the RC lining, and 20 mm was used for the rest of the Lagrangian models). Outflow boundaries were imposed around the perimeter of the ground to simulate an infinite space of surrounding rock.

### 3.2. Blast Scenarios

Based on the potential for terrorist attacks during the life cycle of the tunnel [53], five explosive threats were selected in this section. The potential explosive threats were a suitcase bomb, a compact sedan, a sedan, a cargo van and a delivery truck, which correspond to 23, 227, 454, 1814 and 4536 kg of equivalent TNT weight (*W*), respectively, as shown in Table 11. Numerical simulations of the central explosion and contact explosion at the tunnel crown and tunnel hance for the prototype tunnel entrance were carried out. The purpose was to investigate the dynamic response of the prototype of the tunnel entrance under internal explosions, thus contributing to the determination of the proper TNT charge weight in the following vulnerability study in this paper.

### 3.3. Dynamic Responses of the Prototype of the Tunnel Entrance

**Figure 11:**
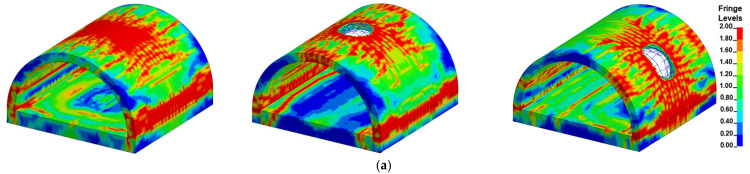

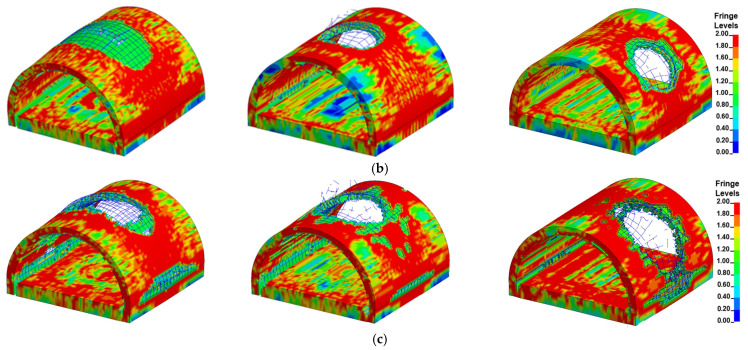
Damage pattern of the prototype of tunnel entrance under given explosive mass (**a**) W = 454 kg; (**b**) W = 1814 kg; (**c**) W = 4536 kg; (The detonation points are: center, tunnel crown and tunnel hance from left to right).

In the present study, the maximum bearing capacity of the prototype tunnel entrance was determined to be 4640 kN via the FE simulation, as stated in Section 2.2. Figure 11 selectively illustrates the damage pattern of the prototype tunnel entrance subjected to the internal explosion with three different TNT charge weights (*W* = 454, 1814, 4536 kg). It is noted that there were three blast scenarios for each TNT charge weight (i.e., the central noncontact explosion, the contact detonation at the tunnel crown and the contact detonation at the tunnel hance). As shown in Table 12, there was no significant reduction in the residual bearing capacity of the tunnel entrance when *W* was 23 kg, implying that the suitcase bomb posed little threat to the tunnel entrance.

As the weight of the explosive increased, the localized punching failure zone and the region of damaged area greatly expanded, which was consistent with the field test results. It is worth noting that for the case of the central noncontact explosion, when the weight of the explosive was less than 454 kg, the tunnel entrance suffered global damage, while local collapse occurred as the weight of the explosive increased to 1814 kg. Under such circumstances, the residual bearing capacity of the tunnel entrance rapidly decreased. As shown in Table 12, when the weight of the explosive reached 4536 kg, the tunnel entrance suffered severe damage regardless of the position of the detonation point, and its corresponding bearing capacity was reduced to 1% of the maximum bearing capacity, implying that failure had occurred in the tunnel entrance.

## 4. Vulnerability Analysis of the Tunnel Entrance

### 4.1. Analysis of Uncertain Issues

**Figure 12:**
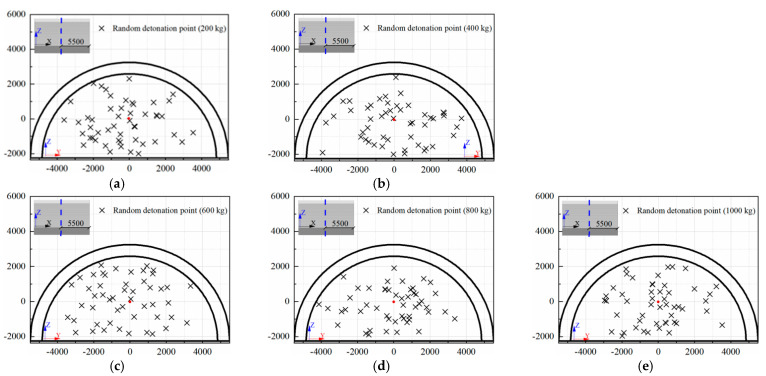
Coordinates of random detonation points (unit: mm): (**a**) *W* = 200 kg, (**b**) *W* = 400 kg, (**c**) *W* = 600 kg, (**d**) *W* = 800 kg, (**e**) *W* = 1000 kg.

For a tunnel structure, there are many random structural issues, such as tunnel dimensions, lining strength, rebar ratio, and burial depth. In addition, the risk of explosion from accidental vehicle explosions and terrorist attacks is associated with a number of uncertainties, such as the explosive type, the location of detonation, and the weight of the explosive [54,55]. In the present study, two main uncertainties were considered, namely the detonation point and weight of the explosive. According to the dynamic response of the prototype of the tunnel entrance under internal explosion in Section 3.3, it was found that when the weight of TNT charge exceeded 1000 kg (i.e., 1814 and 4536 kg), the maximum residual bearing capacity of the tunnel entrance was less than 13.6% of the initial bearing capacity. Under such circumstances, the tunnel entrance seemed to suffer severe failure. Therefore, in the following vulnerability analysis on the tunnel entrance under internal explosion, the case of TNT charge weight beyond 1000 kg was not considered. After several FE simulation trials, in this section, a total of five explosive weights (*W*) were taken into account, which were 200, 400, 600, 800 and 1000 kg. It is further assumed that the internal explosion occurred 5.5 m away from the tunnel entrance. Then, the Monte Carlo method was utilized in Python to conduct two-dimensional random sampling of the detonation points [56,57]. The sample size for each group of explosives was 50, thus a total of 250 random detonation points were located in the Y-Z plane. Figure 12 illustrates the coordinates of the random detonation points for each explosive weight, in which the red point was the origin of the coordinate. It should be noted that if the distance from the detonation point to the inner wall of the tunnel was less than the dimension of the explosive, it was regarded as a contact explosion scenario. The FE model of the prototype of the tunnel entrance in this section remained the same as that in Section 3.

### 4.2. Damage Criterion

In the existing structural analysis under blast loading, the displacement, angle, ductility ratio and bearing capacity of the reinforced concrete members are usually utilized as the damage criterion [58,59]. However, according to the field observations, the failure mode of the tunnel entrance was not unique under various internal explosion conditions. Under the condition of internal noncontact explosion, the tunnel entrance mainly suffered a global response, while under the condition of internal contact explosion, the tunnel entrance exhibited localized punching failure of the tunnel lining. Hence, the displacement damage index in this study cannot accurately evaluate the damage of the tunnel entrance after an internal explosion. For the tunnel structure, the bearing capacity is usually employed as a basic design parameter, and the degradation degree in the bearing capacity is directly related to the deterioration of the mechanical properties of the tunnel structure. Therefore, it is more appropriate to take the degradation degree of the bearing capacity as the damage index for the tunnel entrance under an internal explosion. In this paper, the damage index *D* of the tunnel entrance under internal explosion was defined as [60,61]:

(5) $$D=1-\frac{F_{r}}{F_{0}}$$

where *F*_0_ is the maximum bearing capacity of the intact tunnel entrance. *F*_r_ is the residual bearing capacity of the blast-damaged tunnel entrance, which can be determined from the FE simulation. In this paper, it is assumed that the damage level for the tunnel entrance after an internal explosion can be divided into four categories based on the degradation degree of the bearing capacity: light damage, moderate damage, severe damage and complete damage [60]. Table 13 gives the threshold values for different damage levels in the tunnel entrance.

### 4.3. Analysis of the FE Simulation Results

**Figure 13:**
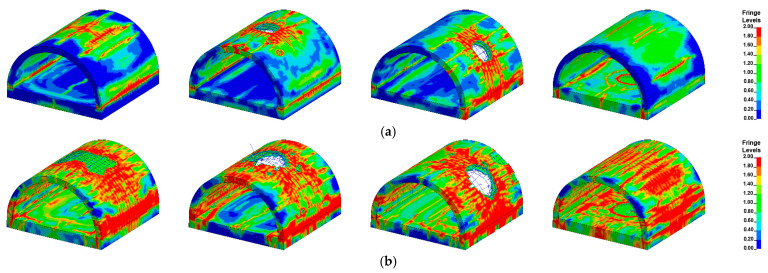

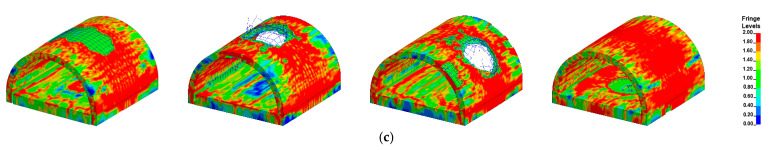
Damage pattern of the prototype of tunnel entrance with different TNT masses: (**a**) *W* = 200 kg; (**b**) *W* = 600 kg; (**c**) *W*= 1000 kg; (From left to right the detonation points are: center, crown, hance and foundation slab).

**Figure 14:**
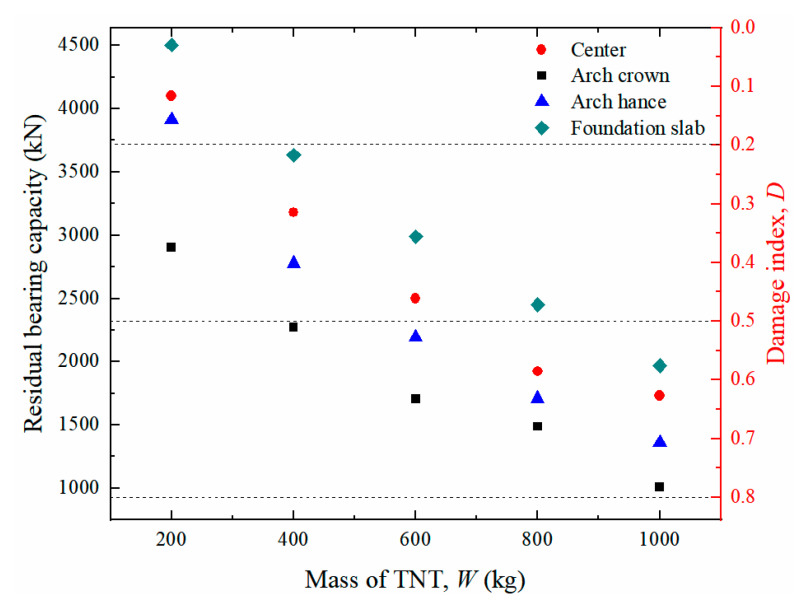
Damage index of the prototype of tunnel entrance with different TNT mass.

Figure 13 displays the typical damage pattern of the prototype of the tunnel entrance under various detonation points for a certain TNT charge weight selected from 250 groups of numerical simulation results. This selection was based on the conditions under which the random detonation points occurred at the tunnel crown, center of the tunnel, tunnel hance and center of the foundation slab. The figure shows that the level of damage in the tunnel entrance was aggravated with increasing TNT charge weight. In particular, for the case of detonation at the foundation slab, a localized crater was formed, and several longitudinal cracks simultaneously developed at the tunnel crown, hance and foot. The residual bearing capacity of the tunnel entrance for each case is presented in Figure 14. It can be seen that the deterioration of the residual bearing capacity of the tunnel entrance was worse for the case of contact explosion at the tunnel crown, whereas it was lighter for the case of contact explosion at the foundation slab, indicating that the tunnel crown was the weakest position of the tunnel entrance. In addition, for explosive weights ranging from 200 kg to 600 kg, the tunnel entrance mainly exhibited light to moderate damage, except for the case of contact detonation at the tunnel crown. When the explosive weight increased from 600 kg to 800 kg, the tunnel entrance damage gradually evolved from moderate to severe. When the explosive weight reached 1000 kg, severe damage to the tunnel entrance was observed regardless of the position of the explosion in the tunnel.

### 4.4. Vulnerability Analysis of the Tunnel Entrance under Internal Explosion

**Figure 15:**
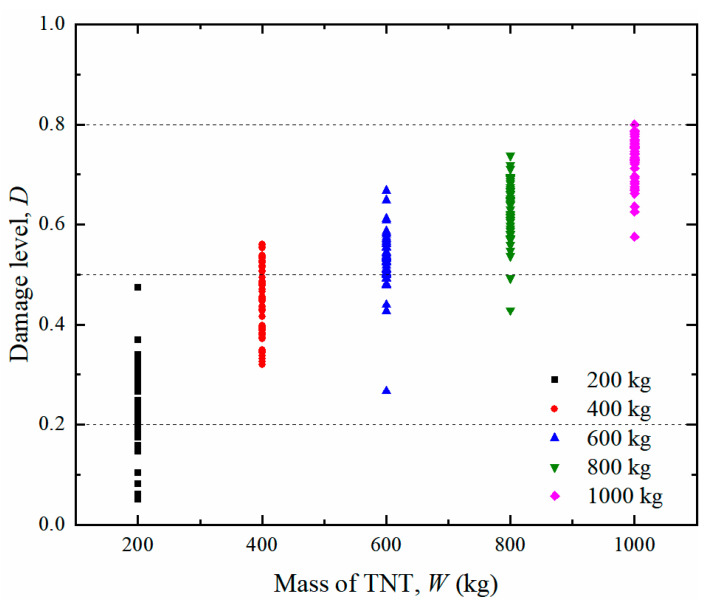
Sample data distribution of *W* and *D*.

The residual bearing capacities were calculated for all the post blast tunnel entrances generated in Section 4.1. Thus, a series of scatter points was obtained, as shown in Figure 15. It is noted that each point in the figure represents the damage level for the tunnel entrance under different detonation points. The three horizontal dashed lines correspond to three damage levels.

**Figure 16:**
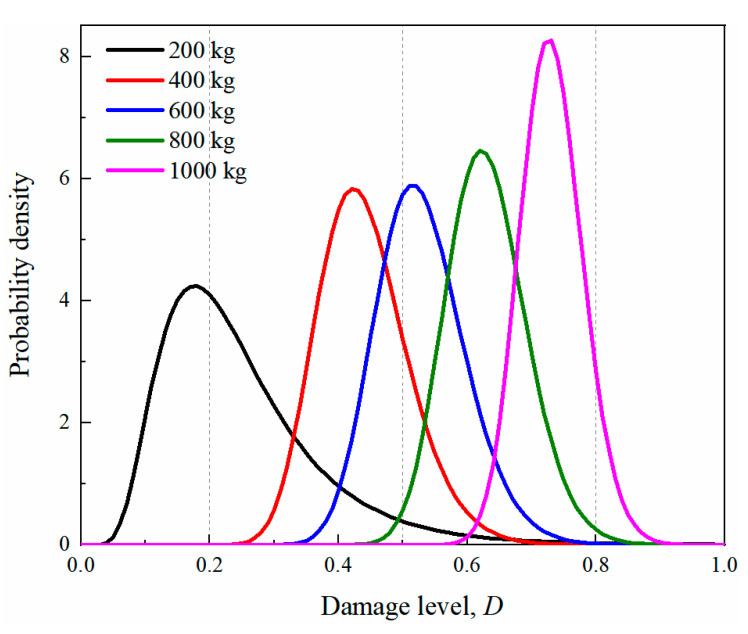
Probability density function of damage index for the tunnel under different equivalence of explosives.

In the present study, it is assumed that the damage index of the sample data under the same explosive weight follows a lognormal distribution [56,62,63,64]. Therefore, the probability density function of the damage index of the tunnel entrance can be further expressed as the lognormal probability density function. The corresponding logarithmic mean and logarithmic standard deviation of the five TNT charge weights are reported in Table 14. From the table, it is shown that with the increase in explosive weights, the mean of the function gradually increased. Based on the data in the table, the probability density function curves of the damage level for different explosive weights are plotted in Figure 16. The dashed lines in the figure represent the threshold value for the corresponding damage level. From the figure, it is observed that the probability density curve shifted to the right as the explosive weight increased, suggesting that the damage to the tunnel entrance became more severe. For an internal explosion with a charge weight of 200 kg, the tunnel entrance suffered light or moderate damage, while for the 1000 kg charge weight, severe or even complete damage of the tunnel entrance occurred.

**Figure 17:**
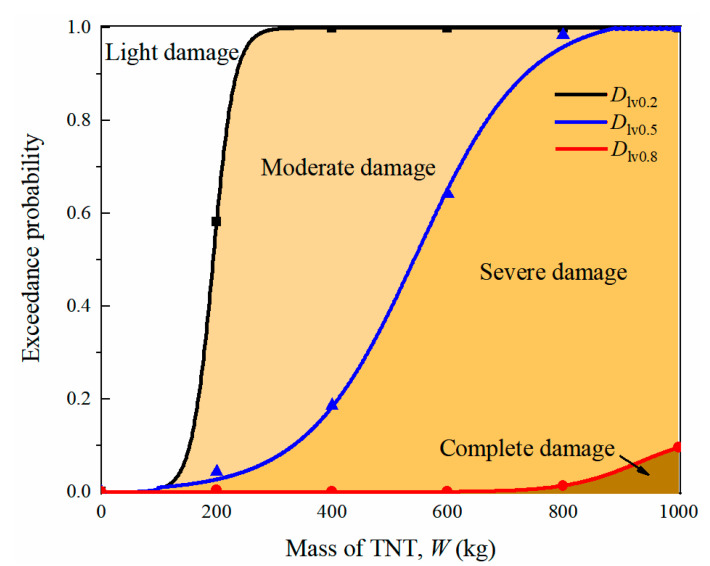
Vulnerability diagram of the prototype of tunnel entrance.

Figure 17 plots the vulnerability curves of the prototype tunnel entrance under internal explosion, in which the horizontal coordinate is the explosive weight and the vertical coordinate represents the exceedance probability for the tunnel entrance under internal explosion. The plane in the figure was divided into four regions (light damage, moderate damage, severe damage and complete damage) by the three curves *D*_lv0_._2_, *D*_lv0_._4_ and *D*_lv0_._6_. From the figure, it is observed that when the explosive weight was greater than 300 kg, the probability of the tunnel entrance exceeding light damage reached 1.0, while the corresponding probability for moderate damage was 0.08. At the same time, the enclosed areas of moderate and severe damage were greater than the other areas, indicating that the prototype of the tunnel entrance was dominated by moderate and severe damage for TNT charge weights ranging from 200 kg to 1000 kg. In addition, the area enclosed by *D*_lv0_._8_ and the coordinate axis were small, implying a low probability of complete damage to the tunnel entrance for explosive weights less than 1000 kg.

## 5. Conclusions

In this paper, an FE model of a tunnel entrance under internal explosion was developed and validated based on field blast tests and laboratory static loading tests. The validated FE model was then employed to conduct a probabilistic vulnerability analysis to quantify the probability of damage to the tunnel entrance under internal explosion. The uncertainty of explosive resources was considered. The residual bearing capacity of the tunnel entrance after blasting was adopted as the damage criterion for damage assessment. The vulnerability curves corresponding to various damage levels were then developed based on the stochastic data from the probabilistic vulnerability analysis. The following conclusions were obtained:

(1) When the tunnel entrance was under the action of contact explosion, a punching failure zone appeared at the location of the detonation point, and a large number of cracks radially developed around the crater, indicating that the tunnel entrance had suffered local damage. When the detonation point was in the center of the tunnel, the tunnel entrance exhibited global damage. Under the same TNT charge weight, the residual bearing capacity of the tunnel entrance after contact explosion at the tunnel crown was the lowest among all the blast scenarios. It can be concluded that damage occurring at the tunnel crown would significantly reduce the bearing capacity and stiffness of the tunnel entrance.
(2) The numerical results of the finite element (FE) model were in good agreement with the actual measurements from the blast and static loading tests in terms of reflected overpressure, damage pattern and residual bearing capacity. The validated FE model was able to precisely predict the damage mode and residual bearing capacity of the post blast tunnel entrance.
(3) For the central noncontact explosion scenario, when the charge weight (*W*) was less than 454 kg, the tunnel entrance suffered global damage, and its corresponding residual bearing capacity dropped to 63% of the maximum bearing capacity. As *W* increased to 1814 kg, the tunnel entrance seemed to collapse. However, when *W* reached 4536 kg, the tunnel entrance suffered severe damage regardless of the position of the detonation point. In addition, when the detonation point was located at the foundation slab, the degradation degree of the tunnel entrance was minimal, but the opposite result was observed when the contact explosion occurred at the tunnel crown, demonstrating that the tunnel crown was the vulnerable position for the tunnel entrance.
(4) When the charge weight (*W*) was in the range of 0–100 kg, the probability of damage to the tunnel entrance increased rapidly. As the charge weight increased past 300 kg, the tunnel entrance’s probability of slight damage reached 1.0. When *W* varied from 200 to 1000 kg, the damage to the tunnel entrance shifted from moderate to severe. However, the tunnel entrance’s probability of complete damage was less than 10% when the TNT charge weight did not exceed 1000 kg. The established vulnerability diagram can be used for probabilistic assessment of the damage level in tunnel entrances subjected to various charge weights or detonation points.
(5) The recommendation for the blast-resistant design of the tunnel in this paper is that the tunnel arch should be the most emphasized part in the reinforcement for the entire structure. In addition, for daily transportation, the threshold value of the TNT equivalent (*W*_t_) of hazardous articles for carrying is 100 kg. When *W*_t_ is less than 100 kg, the vehicles can be permitted to transport. When *W*_t_ exceeds 100 kg, it is recommended for vehicles to pass through the tunnel in batches. The accumulation of *W*_t_ up to 800 kg in the tunnel is strictly prohibited.

## Figures and Tables

**Table 1:** Similarity ratio for the scaled blast test.

Quantity	Symbol	Similarity Factor
Scaling factor	*λ_r_*	1/5.5
TNT mass density	*λ_ρ_*	1
Sound velocity in air	*λ_c_*	1
Time	*λ_t_ = λ_r/_λ_c_*	1/5.5
Blast pressure	*λ_p_ = λ_ρ_ λ_c_* ^2^	1
TNT mass	*λ_M_ = λ_p_ λ_r_ λ_t_* ^2^	1/166.4
Outer radii	*λ_or_ = λ_r_*	1/5.5
Inner radii	*λ_ir_ = λ_r_*	1/5.5
Lining thickness	*λ_lt_ = λ_r_*	1/5.5
Reinforcement ratio	*λ_rr_*	1
Strength	*λ_fc_ = λ_p_*	1
Strain	*λ_ε_*	1

**Table 2:** Program of the field blast test.

Case	TNT Charge (kg)	Position of Explosion in the Tunnel	Schematic	
			Cross-Section	Longitude
B1	2	Center	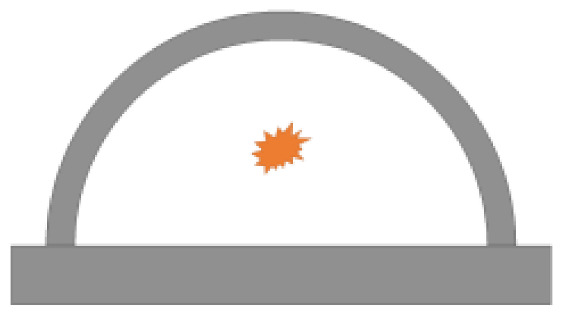	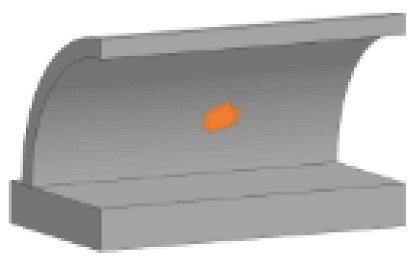
B2	2	Top	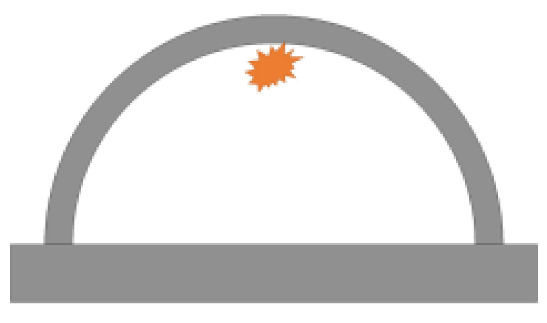	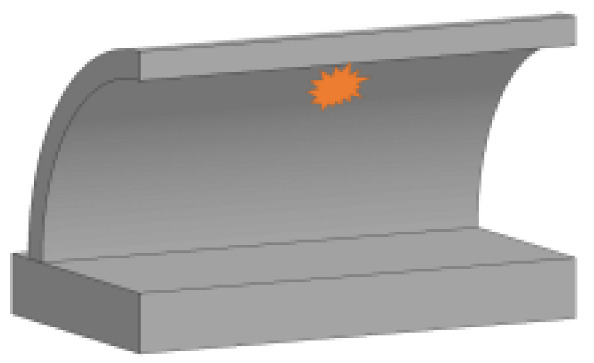
B3	2	Hance	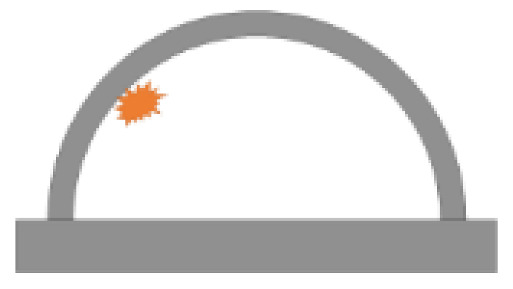	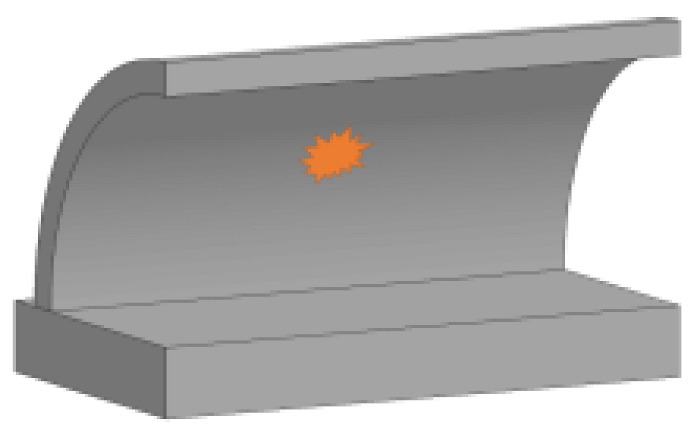

**Table 3:** Test program.

Case	Specimen No.	Position of Explosion
B0	Specimen 4	Intact specimen
B1	Specimen 1	Center
B2	Specimen 2	Top
B3	Specimen 3	Hance

**Table 4:** Key parameters in the KCC model for the tunnel lining and foundation slab.

Tunnel Lining				Foundation Slab			
Parameter	Value	Parameter	Value	Parameter	Value	Parameter	Value
*f_c_* (MPa)	24.7	*a*_1_	0.4463	*f_c_* (MPa)	37.4	*a*_1_	0.4463
*f_t_* (MPa)	2.551	*a*_2_	0.0033	*f_t_* (MPa)	3.363	*a*_2_	0.0022
*w_c_* (mm)	30	*a*_1f_	0.4417	*w_c_* (mm)	30	*a*_1f_	0.4417
*h* (mm)	15	*a*_0y_	5.5130	*h* (mm)	15	*a*_0y_	8.3480
*b*_1_	3.13	*a*_1y_	0.6250	*b*_1_	2.73	*a*_1y_	0.6250
*b*_2_	1.27	*a*_2f_	0.0048	*b*_2_	1.32	*a*_2f_	0.0032
*a*_0_	7.3010	*a*_2y_	0.0104	*a*_0_	11.06	*a*_2y_	0.0069

**Table 5:** Pressure versus volume strain.

Volume Strain (ln*V*)	0	0.104	0.161	0.192	0.224	0.246	0.271	0.283	0.290	0.40
**Pressure (MPa)**	0	8	16	24	48	80	160	240	320	1640

**Table 6:** Key parameters of the soil layer.

Density *ρ* (kg/m^3^)	Shear Modulus *G* (MPa)	Bulk Modulus for Unloading *K* (MPa)	*a*_0_ (Pa^2^)	*a*_2_ (Pa)	*a*_3_
1820	76.01	87.87	0	0	0.47

**Table 7:** Key parameters of air and TNT [33,36,50,51].

Air				TNT			
Parameter	Value	Parameter	Value	Parameter	Value	Parameter	Value
*ρ* (kg/m^3^)	1.29	*C*_4_	0.4	*ρ* (kg/m^3^)	1630	*R*_1_	4.15
*C*_0_	0	*C*_5_	0.4	*D* (m/s)	6930	*R*_2_	0.95
*C*_1_	0	*C*_6_	0	*P_CJ_* (MPa)	21,000	*ω*	0.3
*C*_2_	0	*E*_0_ (J/m^3^)	0.25	*A* (MPa)	374,000	*E*_0_ (J/m^3^)	7 × 10^9^
*C*_3_	0			*B* (MPa)	3230		

**Table 8:** Comparison of the reflected overpressure and acceleration between the test and simulation.

Case	Peak Reflected Overpressure (MPa)						
B1	Sensor No.	RP_7_	RP_8_	RP_9_	RP_10_	RP_11_	RP_12_
	Test	0.93	-	-	1.29	1.26	0.93
	Simulation	0.60	0.51	0.50	1.22	1.47	1.30
	Deviation (%)	−35.5	-	-	−5.4	16.7	39.8
B2	Sensor No.	-	-	-	RP_4_	RP_5_	RP_6_
	Test	-	-	-	2.75	2.61	2.40
	Simulation	-	-	-	2.56	2.25	1.55
	Deviation (%)	-	-	-	−6.9	−13.8	−35.4
B3	Sensor No.	RP_7_	RP_8_	RP_9_	RP_10_	RP_11_	RP_12_
	Test	0.84	1.03	0.68	1.58	1.46	0.57
	Simulation	1.65	1.09	0.83	1.69	0.97	0.33
	Deviation (%)	96.4	5.8	22.1	7.0	−33.6	−42.1

**Table 9:** Size of punching failure zone (unit: mm).

Case	Test				Simulation				Average Deviation
	Inner Face		Outer Face		Inner Face		Outer Face		
	d1	d2	d1	d2	d1	d2	d1	d2	
B2	500	560	530	580	540	600	580	610	7.44%
B3	500	400	600	660	550	448	630	710	8.64%

**Table 10:** Bearing capacity of the specimens between test and simulation.

Case	Specimen No.	Bearing Capacity (kN)		Deviation
		Test	Simulation	
B0	Specimen 5	600.00	585.00	2.5%
B1	Specimen 2	531.64	510.69	3.9%
B2	Specimen 3	454.21	411.52	9.3%
B3	Specimen 4	573.39	507.00	11.5%

**Table 11:** Mass of the potential explosion threats.

Potential Explosion Threats	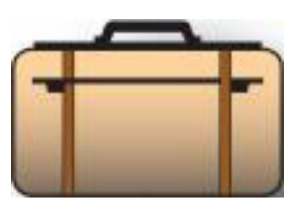	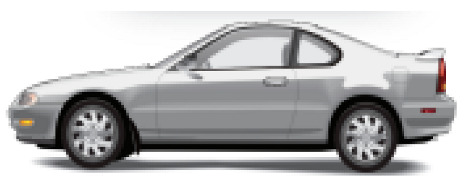	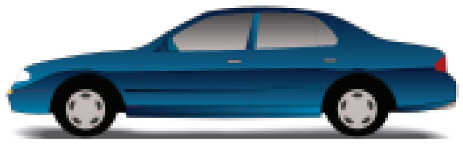	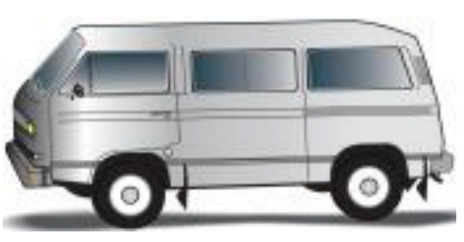	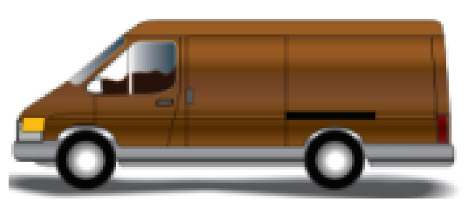
	Suitcase	Compact Sedan	Sedan	Cargo Van	Delivery Truck
TNT equivalent (kg)	23	227	454	1814	4536

**Table 12:** Residual bearing capacity of the prototype of tunnel entrance with given explosive mass.

	Residual Bearing Capacity (kN)		
Position of the Detonation Point	Center	Tunnel Crown	Tunnel Hance
*W* = 23 kg	4598	4370	4452
*W* = 227 kg	3858	2540	3671
*W* = 454 kg	2954	2005	2413
*W* = 1814 kg	330	254	631
*W* = 4536 kg	42.4	20.1	41.8

**Table 13:** Threshold value for different damage levels of the tunnel.

*D*	Damage Levels
0–0.2	Light damage
0.2–0.5	Moderate damage
0.5–0.8	Severe damage
0.8–1	Complete damage

**Table 14:** Lognormal mean and standard deviation under different equivalence of explosives.

*M*/kg	Lognormal Mean (*µ_ln_*)	Lognormal Standard Deviation (*σ_ln_*)
200	−1.510	0.477
400	−0.835	0.160
600	−0.646	0.130
800	−0.466	0.099
1000	−0.315	0.066

## Data Availability

Not applicable.
